# Is essential newborn care provided by institutions and after home births? Analysis of prospective data from community trials in rural South Asia

**DOI:** 10.1186/1471-2393-14-99

**Published:** 2014-03-07

**Authors:** Christina Pagel, Audrey Prost, Munir Hossen, Kishwar Azad, Abdul Kuddus, Swati Sarbani Roy, Nirmala Nair, Prasanta Tripathy, Naomi Saville, Aman Sen, Catherine Sikorski, Dharma S Manandhar, Anthony Costello, Sonya Crowe

**Affiliations:** 1 Clinical Operational Research Unit, University College London, 4 Taviton Street, London WC1H 0BT, UK; 2 Institute for Global Health, University College London, 30 Guilford Street, London WC1N 1EH, UK; 3 Perinatal Care Project, Diabetic Association of Bangladesh, 122 Kazi Nazrul Islam Avenue, Dhaka 1000, Bangladesh; 4 Ekjut, Plot 556B, Potka, Chakradharpur, West Singhbhum, Jharkhand, India; 5 Mother Infant Research Activities (MIRA), YB Bhavan, Thapathali, GPO Box 921, Kathmandu, Nepal

## Abstract

**Background:**

Provision of essential newborn care (ENC) can save many newborn lives in poor resource settings but coverage is far from universal and varies by country and place of delivery. Understanding gaps in current coverage and where coverage is good, in different contexts and places of delivery, could make a valuable contribution to the future design of interventions to reduce neonatal mortality. We sought to describe the coverage of essential newborn care practices for births in institutions, at home with a skilled birth attendant, and at home without a skilled birth attendant (SBA) in rural areas of Bangladesh, Nepal, and India.

**Methods:**

We used data from the control arms of four cluster randomised controlled trials in Bangladesh, Eastern India and from Makwanpur and Dhanusha districts in Nepal, covering periods from 2001 to 2011. We used these data to identify essential newborn care practices as defined by the World Health Organization. Each birth was allocated to one of three delivery types: home birth without an SBA, home birth with an SBA, or institutional delivery. For each study, we calculated the observed proportion of births that received each care practice by delivery type with 95% confidence intervals, adjusted for clustering and, where appropriate, stratification.

**Results:**

After exclusions, we analysed data for 8939 births from Eastern India, 27 553 births from Bangladesh, 6765 births from Makwanpur and 15 344 births from Dhanusha. Across all study areas, coverage of essential newborn care practices was highest in institutional deliveries, and lowest in home non-SBA deliveries. However, institutional deliveries did not provide universal coverage of the recommended practices, with relatively low coverage (20%-70%) across all study areas for immediate breastfeeding and thermal care. Institutions in Bangladesh had the highest coverage for almost all care practices except thermal care. Across all areas, fewer than 20% of home non-SBA deliveries used a clean delivery kit, the use of plastic gloves was very low and coverage of recommended thermal care was relatively poor. There were large differences between study areas in handwashing, immediate breastfeeding and delayed bathing.

**Conclusions:**

There remains substantial scope for health facilities to improve thermal care for the newborn and to encourage immediate and exclusive breastfeeding. For unattended home deliveries, increased handwashing, use of clean delivery kits and basic thermal care offer great scope for improvement.

## Background

Up to 1.2 million newborns [1] and 83 000 new mothers [2] die every year in South Asia, representing over half the global number of neonatal deaths and one third of maternal deaths. The World Health Organization (WHO) has published guidelines for Essential Newborn Care (ENC) including protective practices before, during, and after birth [3]. Several South Asian countries have increased the number of women giving birth in institutions or at home with a skilled birth attendant (SBA) [4-6]. Increasing access to skilled attendance at birth can reduce both maternal and neonatal mortality, but in resource-poor areas, having a skilled birth attendant or an institutional delivery may not guarantee full provision of WHO-recommended essential newborn care [7-12]. Increased access to some components of newborn care for women giving birth at home without a SBA can also be an effective strategy to reduce neonatal morbidity and mortality [5,6,13], for instance through the use of clean delivery kits [14,15]. Both strategies are based on the knowledge that increasing the coverage of essential newborn care practices in South Asia is essential to achieving the millennium development goals for child survival [16-18].

Lawn *et al*. highlighted the importance of using local data to guide program implementation [8]. Where national policies aim to increase institutional deliveries and/or deliveries at home with an SBA, understanding the nature of care available for each type of delivery provides an opportunity to improve the care received by women and newborns. Similarly, understanding what happens during home deliveries without an SBA which are common in rural areas [19,20], could provide insights into how to promote safer practices for these women.

In 2011, some Demographic Health Surveys (DHS) began to collect data on newborn care, including in Bangladesh and Nepal, underscoring the importance of measuring improvements in newborn care practices to improve both neonatal and overall child survival [21,22]. The DHS surveys provide important information, but data on newborn care are only collected for women giving birth at home, and women can be asked for recollections going back a year or more, which makes the information collected susceptible to recall bias. Moreover, while it is very important to understand what happens during and after home births, we should not assume that care received in an institution necessarily meets the standards of WHO-recommended essential newborn care. There can be too few personnel trained in ENC within institutions [23], and even trained personnel do not necessarily recommend or practice all components of ENC [17,24].

There have been many valuable reports on the coverage of ENC in rural South Asia, but these tend either to not report coverage by different types of delivery [25-28], or to cover relatively few births [18,29,30] and/or relatively few practices [17,25,29] and most consider only one geographical area. Reported ENC coverage varies greatly between these studies but all suggest that coverage is far from universal, particularly among home births. Examining the observed coverage of ENC for different areas and different delivery types can provide important information to those planning and delivering interventions to improve newborn and maternal outcomes in South Asia, whether at home or in institutions. The CHERG working group on Improving Coverage Measurement has also recently called for more evidence of the actual coverage achieved within different populations and types of delivery [31,32].

In this paper, we use data from four community cluster randomised controlled trials in rural areas of Nepal, Bangladesh and Eastern India [33-36], covering over 55 000 deliveries, to estimate the proportion of women in each study area receiving a wide range of components of ENC in the intrapartum period and the first 24 hours after birth for deliveries at home without an SBA, at home with an SBA, and in a health facility. Achieved coverage within these four study areas is worth presenting in a single paper since all study areas are in regions with relatively poor access to health care where most women give birth at home, but are nonetheless different culturally, geographically and contextually in terms of state provision of care. We hope that the information presented here will highlight gaps in current coverage within study areas, but also show where coverage is good, thus potentially contributing to the future design of interventions to improve coverage both within each area and throughout South Asia as a whole.

## Methods

### Trials from which data originated

We used data from four cluster randomised controlled trials conducted in Nepal [33,34] (lowlands (2006-11) and middle hills regions (2001-8)), Bangladesh [35,37] (Bogra, Maulvibazaar and Faridpur Districts, 2005-9) and Eastern India [36] (Odisha and Jharkhand states, 2005-8). The trials were all based in rural, underserved communities and tested the implementation of a four-phase participatory learning and action cycle with women’s groups in intervention villages, led by a local female facilitator. In each cluster, fieldworkers sought to identify either all pregnancies (Makwanpur, Nepal) [33], or all recent deliveries [34-36] during the trial period and enrol mothers and their newborn infants into the study. In the Dhanusha (Nepal) trial [34], information was collected on all identified births during the baseline period and then a maximum of 10 per cluster per month during the trial period from April 2007 to April 2011. The estimated study populations ranged from 228 000 (Eastern India) to 670 000 (Dhanusha, Nepal). The key characteristics of each study are summarised in Table 1. Detailed descriptions of the trial areas, data collection and data quality are available in papers specific to each trial [33-36].

**Table 1:** Brief descriptions of the study populations in the four areas

**Project partner**	**Perinatal Care Project (PCP)**	**Ekjut**	**MIRA (Makwanpur)**	**MIRA (Dhanusha)**
Study setting	Rural Bangladesh	Rural Eastern India	Rural Nepal (middle hills)	Rural Nepal (lowlands)
Location of clusters	Bogra, Maulvibazaar and Faridpur Districts	West Singhbhum and Saraikela Districts (Jharkhand); Keonjhar District (Odisha)	Makwanpur District, central region middle hills.	Dhanusha District, plains of Nepal.
Period for which data are included (dates of birth)	1^st^ Feb 2005–31^st^ Dec 2009	1^st^ July 2005–30^th^ June 2008	1^st^ November 2001–31^st^ October 2004 (Phase 1)	1^st^ September 2006–13^th^ April 2011
			1^st^ November 2004–31^st^ October 2008 (Phase 2)	
Total estimated study population	478 000	228 000	400 000	670 000
Design	Two-by-two factorial cluster RCT which ran from 1^st^ Feb 2005 to 31^st^ Dec 2007. A new trial took place using the same clusters from 2009-2011. Data continued to be collected in all clusters from 1^st^ Jan 2008 to 31 Dec 2009 and continued into the new trial period (not included here).	Cluster RCT	Cluster RCT	Two-by-two factorial cluster RCT
Stratification	By district (3 strata)	By district (3 strata)	None	By cluster size (2 strata)
Cluster characteristics	Villages making up a union	8-10 villages with most residents classified as tribal or other backward class	Village development committee	Village development committee
Total number of clusters (number included in this study)	18 (9)	36 (18)	24 (12) (Phase 1)	60 (30)
			30 (6) (Phase 2–former control clusters became intervention clusters and 6 new control clusters recruited)	
Annual births sampled per cluster (after exclusions): Mean (SD)	596 (119)	171 (38)	115 (70)	104 (17)
Approximate cluster population	28 000	6400	4000	8000

In this study, we use data only from the control clusters in order to gain a better understanding of the components of essential newborn care received by women in these communities in the absence of the intervention women’s groups. Although the Makwanpur (Nepal) trial was conducted in two phases, we combined data from both phases since only control data are used and phase 2 followed directly from phase 1. The Dhanusha (Nepal) and Bangladesh trials were factorial design trials. The Dhanusha (Nepal) trial additionally tested an intervention to identify and treat neonatal sepsis. However, given our focus on ENC, we believe this additional intervention would not affect the components of essential newborn care that the women received. The Bangladesh trial additionally tested an intervention on training traditional birth attendants (TBA) in the use of a bag and mask to treat neonatal asphyxia. This intervention would certainly not have had any impact on the components of essential newborn care received by women giving birth at home with an SBA or in an institution. We note that TBAs in all arms of the trial received training in essential newborn care, the only difference between arms being in the additional training in the use of a bag and mask [35]. Given that we do not consider resuscitation practices or mortality in this study (see Table 2), we treat all non-women’s groups clusters as control clusters.

**Table 2:** Care practices included in the study

**Care practice**	**Ekjut (Eastern India)**	**PCP (Bangladesh)**	**Makwanpur (Nepal)**	**Dhanusha (Nepal)**
**Antepartum hygienic care practices**				
Attendant washed hands before delivery	X	X	X	X
Clean Delivery Kit used	X	X	X	X
Attendant used disposable gloves	X	X		
Plastic sheet used	X	X		
**Intrapartum and postnatal cord care**				
Thread/clamp used during delivery	X	X	X	X
Cord tied with boiled thread	X	X		
Cord cut with new/sterile blade	X	X	X^†^	X^†^
Nothing/only antiseptic applied to cord stump	X	X	X*	X*
**Postnatal newborn care**				
Clean cloth used for wrapping	X	X		
Immediate wiping (within 10 minutes)	X	X		
Skin-to-skin contact between mother and baby within 30 minutes	X	X		
Thermal care (wrapping or skin-to-skin contact within 10 minutes)	X	X	X**	X**
Delayed bathing (baby not bathed for at least 6 hours)	X	X	X	X^‡^
Colostrum not discarded			X	X
No pre-lacteal feed (breast milk first food)	X	X	X	X
Immediate breastfeeding ( within 1 hour)	X	X	X	X
Only breast milk in first 24 hours	X	X		

“X” indicates whether relevant information about the care practice is available for that study.

†For Eastern India and Bangladesh, data were available both on whether a new blade was bought for cord cutting and whether the implement used for cutting (whether a new blade or not) had been boiled prior to use. For the Nepal areas, there was no information on whether the blade was new and only whether the cord had been cut with a boiled blade.

*In both Nepal areas, data were available on whether nothing was applied to the cord or ‘medicine/dettol’.

**For Eastern India and Bangladesh, thermal care was defined as “either wrapped or skin-to-skin contact within 10 minutes”. Questions on skin-to-skin were not asked in Nepal and so thermal care was defined as wrapping “Immediately” (Dhanusha) or “within 10 minutes” (Makwanpur).

‡For Dhanusha, detailed timing information was not available and so delayed bathing was defined as “not bathed in 24 hours”.

### Care practices

We used the WHO Guide to Essential Newborn Care [3] to identify best care practices in the intrapartum and immediate postnatal period that corresponded to data collected during the trials. The care practices identified by the World Health Organisation are based on best available evidence and represent “a common understanding between WHO, UNFPA, UNICEF, and the World Bank of key elements of an approach to reducing maternal and perinatal mortality and morbidity” [3]. We thus considered these to be the best definitions available for trying to understand the provision of essential newborn care in South Asia. Where the trials did not have exact matches to the WHO definitions, we used the closest match. Practices identified for each trial are given in Table 2, where a cross indicates that data on a given practice were available. The WHO recommendation and detailed questions asked for each practice in each trial are given in Additional file 1. We arranged the identified practices into three groups: hygienic care practices just before delivery; intrapartum and postnatal cord care; postnatal newborn care.

### Data analysis

We excluded records for: mothers in the women’s group arm; mothers who migrated out of the study area; all multiple births except the first born child (to avoid counting practices for the same delivery multiple times); maternal deaths in the antenatal period, and infants who died in utero. Control data were analysed separately for each trial.

Each remaining birth was allocated to one of three delivery types: home birth without an SBA (home non-SBA), home birth with an SBA (home SBA) or institutional delivery. Deliveries were identified as institutional if the women gave birth in one of: government or private hospital, primary health care centre, health post, government health centre, charitable hospital or a Maternal and Child Welfare Centre. For home births, a birth was identified as an SBA birth if the main attendant was a doctor, a nurse, a government health worker, or an auxiliary nurse midwife. We note that an institutional delivery is not necessarily an SBA delivery, but we felt that exploring what happened in institutions (regardless of SBA attendance) would be the most useful information. Mothers who were recorded as having transferred to an institution during delivery were counted as having had an institutional delivery for the post-delivery practices. Data where the delivery type was missing or could not be determined were excluded. The exact definitions used for each delivery type in each study area are given in Additional file 2. Consistent with terms used by the World Health Organization [38], traditional birth attendants (TBAs) were not considered as skilled birth attendants for this study. We note that 15% of all deliveries in the control arm in Bangladesh were by TBAs given four days of training in safer care practices for a trial testing use of bag and mask for neonatal resuscitation [35,39]. For this reason, and because the role and training of TBAs varies between countries, we present a secondary analysis of care practices within home non-SBA deliveries separated by whether the main attendant was a TBA.

We calculated the observed proportion of births receiving each care practice with 95% confidence intervals for each study area and by delivery type, adjusting for the clustered design and, where appropriate, stratification (Eastern India, Bangladesh and Dhanusha (Nepal)). We excluded records for which information on the care practice of interest was missing from that particular analysis. For post-delivery care practices, we also excluded stillbirths and intrapartum maternal deaths since these events could realistically have changed the course of the delivery. All analyses were conducted in Stata/IC 12.1 (StataCorp LP).

We note that when adopting logistic regression with random effects to estimate the proportion for each care practice/delivery type combination, more than 10% of combinations had a quadrature check indicating that the fitted model was not reliable. We therefore adjusted for clustering using cluster-level analyses following the methods in Hayes [40] for all combinations of care practice/delivery type. While resulting in slightly larger confidence intervals, these analyses were more robust and allowed us to use the same method for all combinations.

We did not perform statistical tests for significance in differences of coverage of care practices between delivery types or study areas, as we did not aim to test any specific hypotheses about coverage. While we do expect differences between areas and delivery types, we do not believe that a p-value of significance of these differences would provide particularly useful information. In an ideal world, coverage of ENC would be 100% for all delivery types in all areas. Our aim is to present the situation in the study areas during the study periods, where we know universal coverage was not achieved. Given that the typical coverage for essential newborn care practices in our study areas is between 20-70%, policy is unlikely to be decided on the basis of knowing whether the difference in coverage between two birth practices or two areas is significant, but rather on the achieved coverage for each practice, and the potential for this to be improved within each type of delivery. We hope that the descriptive coverage data will prove useful to those working in these settings, both by highlighting gaps in coverage for different modes of delivery and by showing where coverage is good.

### Ethics

All trials from which data for this study were drawn were approved by the ethics committee of the Institute of Child Health and Great Ormond Street Hospital for Children (UK) and by the following research ethics committees: the ethical review committee of the Diabetic Association of Bangladesh; an independent ethics committee in Jamshedpur, India (Eastern India trial); the Nepal Health Research Council (Dhanusha and Makwanpur, Nepal). All trials were conducted in disadvantaged areas with high levels of female illiteracy; all participants gave consent in writing, by thumbprint or verbally.

## Results

After exclusions, we analysed data from 8939 births from Eastern India, 27 553 births from Bangladesh, 6765 births from Makwanpur (Nepal) and 15 344 births from Dhanusha (Nepal). We note that Makwanpur (Nepal) data includes data from 2001 and so represents a much longer time period than the other trials and the data pre-dates the situation in other sites.

Demographic information about each study area is given in other papers [33-36], but all areas were poor with low levels of maternal literacy (Eastern India: 28%; Bangladesh: 67%; Makwanpur (Nepal): 42%; Dhanusha (Nepal): 28%). We note that levels of maternal education were higher in Bangladesh than the other study areas.

**Figure 1:**
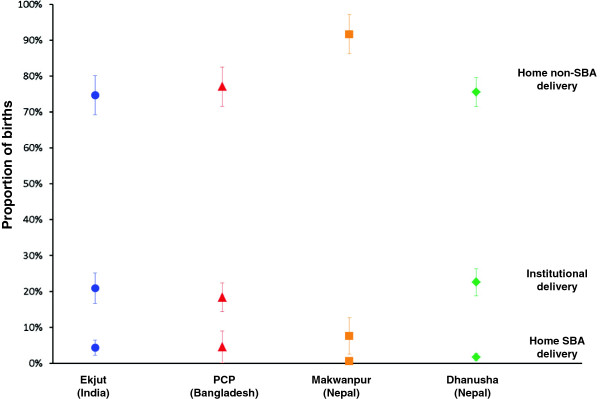
**Proportion of births in each delivery type, for each study area (with 95%confidence intervals).**

The proportions of births for each delivery type in each study are shown in Figure 1. Home non-SBA deliveries were by far the most common across all studies, particularly in Makwanpur (Nepal) where they represented 90% of births. Institutional deliveries represented around 20% of deliveries (except for Makwanpur (Nepal) (7.6%)), while home SBA deliveries were rare in all study areas. The proportion of home non SBA-births where the main attendant was a TBA are given for each area in Table 3.

**Table 3:** Proportion of non-SBA home deliveries that were delivered by a traditional birth attendant

**Study area**	**Proportion of non-SBA home deliveries where the main attendant was a TBA % and 95% confidence interval**
Eastern India	41 [30-51]
Bangladesh	83 [79-87]
Makwanpur (Nepal)	3 [1-4]
Dhanusha (Nepal)	23 [16-29]

**Figure 2:**
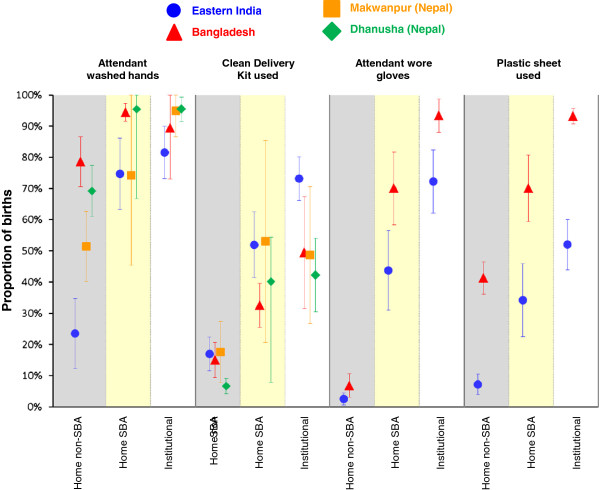
**Proportions of births receiving each antenatal hygienic care practice by study and delivery type with 95%confidence intervals.** Shaded columns are intended to clarify separation between delivery types. We have included a small offset between studies within each delivery type/care practice combination to help show the confidence interval ranges.

**Figure 3:**
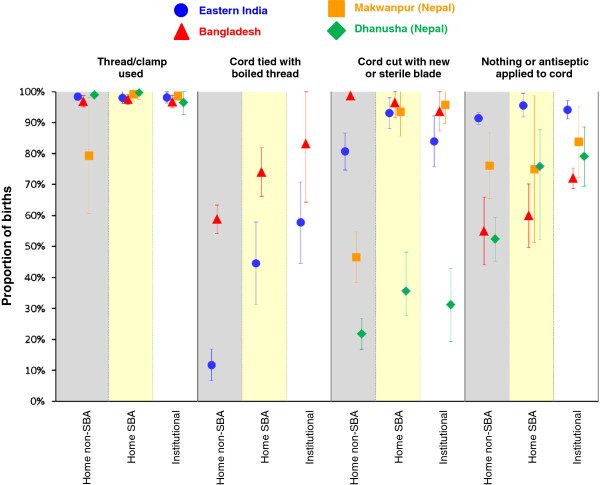
**Proportions of births receiving each intrapartum and postnatal cord care practice by study and delivery type with 95%confidence intervals.** Shaded columns are intended to clarify separation between delivery types. We have included a small offset between studies within each delivery type/care practice combination to help show the confidence interval ranges.

**Figure 4:**
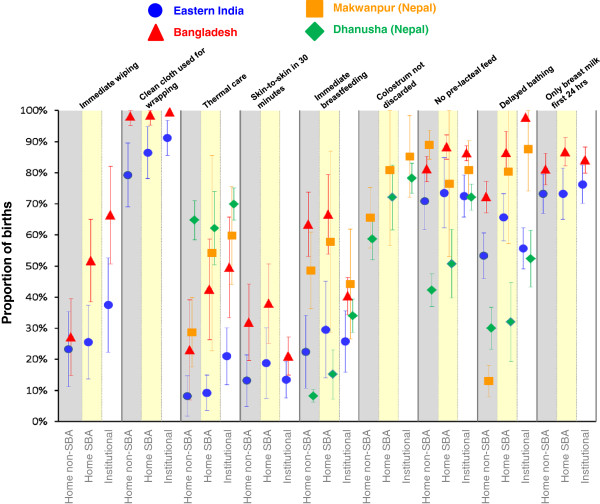
**Proportions of births receiving each postnatal newborn care practice by study and delivery type with 95%confidence intervals.** Shaded columns are intended to clarify separation between delivery types. We have included a small offset between studies within each delivery type/care practice combination to help show the confidence interval ranges.

Figure 2 shows the proportions of deliveries for which mothers received hygienic care just before birth (birth attendant washed hands, used a clean delivery kit, wore gloves, used a plastic sheet). Data on the use of plastic sheets and gloves were only collected in Eastern India and Bangladesh. Figure 3 shows the proportions of deliveries with recommended cord care practices. Neither of the two Nepal studies had data regarding whether a new blade was used to cut the cord, or whether boiled thread was used to tie the cord. Finally, results for the postnatal newborn care practices are shown in Figure 4. Only the Nepal studies had specific data on colostrum and only the Eastern Indian and Bangladesh studies had data regarding use of a clean cloth for wrapping, skin-to-skin contact, immediate wiping and giving only breast milk in the first day.

The solid vertical lines in each figure separate the different care practices, while the light dashed vertical lines separate the different delivery types. We also differentiated the delivery types by shading to aid the reader. Each care practice considered is given at the top of the chart and the different studies are indicated by both colour and shape. We have included a small offset between studies within each delivery type/care practice combination to help show the confidence interval ranges. For completeness, the proportions of deliveries with each component of ENC within each delivery type and area are given in Tables 4 and 5.

**Table 4:** Proportion of deliveries with protective antepartum and intrapartum practices for different delivery types

**Care practice**	**Ekjut (Eastern India)**			**PCP (Bangladesh)**			**Makwanpur (Nepal)**			**Dhanusha (Nepal)**		
**Proportion of antepartum hygienic care practices by delivery type, with 95%confidence intervals (%)**												
**Attendant:**	**Home non-SBA (74.5%of deliveries)**	**Home SBA (4.5%of deliveries)**	**Institution (21%of deliveries)**	**Home non-SBA (77%of deliveries)**	**Home SBA (5%of deliveries)**	**Institution (18%of deliveries)**	**Home non-SBA (91.5%of deliveries)**	**Home SBA (1%of deliveries)**	**Institution (7.5%of deliveries)**	**Home non-SBA (78%of deliveries)**	**Home SBA (1.5%of deliveries)**	**Institution (20.5%of deliveries)**
Washed hands	24 [12-35]	75 [63-86]	82 [73-90]	79 [71-87]	94 [92-97]	89 [73-100]	51 [40-63]	74 [46-100]	95 [87-100]	69 [61-77]	95 [87-100]	95 [91-99]
Used a Clean Delivery Kit	17 [12-22]	52 [41-63]	73 [66-80]	15 [9-21]	33 [25-40]	49 [32-67]	18 [8-27]	53 [21-85]	49 [27-71]	7 [4-9]	40 [26-54]	42 [30-54]
Used disposable gloves	3 [1-5]	44 [31-56]	72 [62-82]	7 [3-11]	70 [58-82]	93 [88-99]						
Used a plastic sheet	7 [4-11]	34 [23-46]	52 [44-60]	41 [36-46]	70 [59-81]	93 [91-96]						
**Proportion of intrapartum and postnatal cord care by delivery type with 95**% **confidence intervals (%)**												
Thread/clamp used during delivery	98 [98-99]	98 [96-100]	98 [96-100]	97 [95-99]	98 [96-99]	97 [95-99]	79 [61-98]	99 [97-101]	99 [97-100]	99 [99-99]	100 [99-100]	96 [93-100]
Cord tied with boiled thread	12 [7-17]	45 [31-58]	58 [45-71]	59 [54-63]	74 [66-82]	83 [64-100]						
Cord cut with new/sterile blade	81 [75-87]	93 [88-98]	84 [76-92]	99 [98-99]	96 [92-100]	94 [87-100]	47 [38-55]	93 [86-101]	96 [90-102]	22 [17-27]	36 [23-48]	31 [19-43]
Nothing or only antiseptic applied to cord stump	91 [90-93]	96 [92-99]	94 [91-97]	55 [44-66]	60 [50-70]	72 [69-75]	76 [66-87]	75 [51-99]	84 [72-95]	52 [45-60]	76 [64-88]	79 [69-89]

Proportion of deliveries with protective antepartum and intrapartum practices for different delivery types: at home with no skilled birth attendance (SBA), at home with SBA and in an institution. Practices are as defined in Table 2.

**Table 5:** Proportion of deliveries with protective postnatal care practices for different delivery types

**Care practice**	**Ekjut (Eastern India)**			**PCP (Bangladesh)**			**Makwanpur (Nepal)**			**Dhanusha (Nepal)**		
**Proportion of postnatal newborn care by delivery type with 95%confidence intervals (%)**												
	**Home non-SBA (74.5%of deliveries)**	**Home SBA (4.5%of deliveries)**	**Institution (21%of deliveries)**	**Home non-SBA (77%of deliveries)**	**Home SBA (5%of deliveries)**	**Institution (18%of deliveries)**	**Home non-SBA (91.5%of deliveries)**	**Home SBA (1%of deliveries)**	**Institution (7.5%of deliveries)**	**Home non-SBA (78%of deliveries)**	**Home SBA (1.5%of deliveries)**	**Institution (20.5%of deliveries)**
Clean cloth used for wrapping	79 [69-90]	86 [78-95]	91 [86-97]	98 [95-100]	99 [95-100]	100 [99-100]						
Immediate wiping (within 10 minutes)	23 [11-35]	26 [14-37]	37 [22-53]	27 [15-39]	52 [39-65]	66 [51-82]						
Skin-to-skin contact between mother and baby within 30 minutes	13 [5-21]	19 [8-30]	13 [8-19]	32 [20-44]	38 [25-51]	21 [15-27]						
Thermal care (wrapping or skin-to-skin contact within 10 minutes)	8 [2-15]	9 [4-15]	21 [12-30]	23 [7-39]	42 [26-59]	50 [33-66]	29 [17-40]	54 [23-86]	60 [44-75]	65 [58-71]	62 [50-74]	70 [65-75]
Delayed bathing (baby not bathed for at least 6 hours)	53 [46-61]	66 [58-73]	56 [49-62]	72 [67-77]	86 [80-93]	98 [97-98]	13 [8-18]	80 [57-104]	88 [74-100]	30 [23-37]	32 [19-45]	52 [43-62]
Colostrum not discarded							66 [56-75]	81 [57-100]	85 [72-98]	59 [52-66]	72 [62-83]	78 [73-83]
No pre-lacteal feed (breast milk first food)	71 [62-80]	74 [62-85]	73 [66-79]	81 [77-85]	88 [84-92]	86 [84-89]	89 [84-94]	76 [53-100]	81 [71-90]	42 [37-47]	51 [40-62]	72 [68-76]
Immediate breastfeeding (within 1 hour)	22 [11-34]	30 [14-45]	26 [16-36]	63 [53-74]	67 [54-79]	40 [34-46]	49 [36-61]	58 [29-87]	44 [27-62]	8 [6-10]	15 [7-23]	34 [29-39]
Only breast milk in first 24 hours	73 [67-80]	73 [65-81]	76 [70-82]	81 [76-86]	87 [82-91]	84 [80-88]						

Proportion of deliveries with protective postnatal care practices for different delivery types: at home with no skilled birth attendance (SBA), at home with SBA and in an institution. Practices are as defined in Table 2.

### Overview of care practices by type of delivery

In all study areas, institutional deliveries had the highest coverage for almost all care practices (Figures 2, 3 and 4, Tables 4 and 5), followed by home SBA deliveries. Unsurprisingly, home non-SBA deliveries had the lowest coverage. However, there are patterns of coverage within each type of delivery that merit further discussion.

### Institutional deliveries

Institutional deliveries did not provide universal coverage of the recommended practices, with relatively low coverage across all study areas for immediate breastfeeding and thermal care (skin-to-skin contact, immediate wrapping and wiping) (Figure 4 and Table 5). Delayed bathing, also a thermal care practice, was high in Bangladesh and Makwanpur (Nepal) but low in the other two study areas. Clean delivery kit use was also low for institutional deliveries (Table 4), but this may reflect protocols that do not require kit use in health facilities. For instance, in Dhanusha, (Nepal) sterile autoclaved equipment rather than kits may be used in health facilities above the health post level. Rates of immediate breastfeeding and early skin-to-skin contact are lower in institutions than for other delivery types in all areas (except immediate breastfeeding in Dhanusha (Nepal)). Institutions in Bangladesh offered the highest coverage for almost all safer care practices except thermal care. Comparing India and Bangladesh, which used very similar questionnaires, there were large differences in the reported practices received in institutions, with mothers who delivered in Indian institutions reporting much lower coverage of disposable glove use, plastic sheet use, cord care practices and thermal care. The only practice where coverage was close to 100% across all study areas was the use of a thread/clamp following delivery.

### Home SBA deliveries

The data for home SBA deliveries are harder to interpret due to small numbers of such births in each cluster leading to large confidence intervals around estimates. However, the general rule holds that coverage is better than in home non-SBA deliveries but worse, if only slightly, than in institutional deliveries. The interesting exception to this rule is around breastfeeding and skin-to-skin practices where coverage tends to be highest for home SBA deliveries.

### Home non-SBA deliveries

The coverage of practices for home non-SBA deliveries varied widely across the different study areas but there are some common features. Across all areas, fewer than 20% of home non-SBA deliveries used a clean delivery kit, the use of plastic gloves was very low (Figure 2, Table 4) and coverage of recommended thermal care was relatively poor (Figure 4, Table 5). There was particularly wide variation between study areas in handwashing (from 24% in Eastern India to 79% in Bangladesh), delayed bathing (from 13% in Makwanpur (Nepal) to 72% in Bangladesh) and immediate breastfeeding (from 8% in Dhanusha (Nepal) to 63% in Bangladesh). As well as large differences between study areas, there is large variability between clusters within study areas for almost all pre-birth hygiene practices and postnatal newborn care practices.

### Home non-SBA deliveries: differences between TBA and non-TBA attended deliveries

Although traditional birth attendants do not (as a group) meet the WHO definitions of skilled birth attendants, many have received some training in ENC practices and many also have the benefit of many years of experience in delivering babies. The use of TBAs by mothers also varies widely by area from only 3% of home non-SBA deliveries in Makwanpur (Nepal) to over 80% of such deliveries in Bangladesh. The breakdown of ENC practices by TBA attendance for home non-SBA deliveries is given in Tables 6 and 7. Practices where there was at least a 10 percentage point difference between TBA and non TBA deliveries are highlighted in bold font. We note that TBAs are not comparable across sites: for instance, most TBAs in Nepal are untrained and within Dhanusha, tend to have low socio-economic status and little formal education. Bearing this in mind, this secondary analysis is included for interest and for additional context for non-SBA home deliveries for each study area. Unsurprisingly, the components of essential newborn care for TBA deliveries differed across study areas.

**Table 6:** proportion of deliveries with protective antepartum and intrapartum practices for mothers who delivered at home with or without a traditional birth attendant

**Care practice**	**Ekjut (Eastern India)**			**PCP (Bangladesh)**			**Makwanpur (Nepal)**			**Dhanusha (Nepal)**		
	**41%of home non-SBA deliveries were by TBA**			**83%of home non-SBA deliveries were by TBA**			**3%of home non-SBA deliveries were by TBA**			**23%of home non-SBA deliveries were by TBA**		
**Proportion of antepartum hygienic care practices in non-SBA home deliveries with 95%confidence intervals (%)**												
	**No TBA**	**TBA**	**All non-SBA home**	**No TBA**	**TBA**	**All non-SBA home**	**No TBA**	**TBA**	**All non-SBA home**	**No TBA**	**TBA**	**All non-SBA home**
Attendant washed hands before delivery	**16 [9-24]**	**36 [24-49]**	**24 [12-35]**	**58 [41-75]**	**83 [76-90]**	**79 [71-87]**	**50 [38-61]**	**92 [83-100]**	**51 [40-63]**	71 [63-79]	66 [57-76]	69 [61-77]
Clean Delivery Kit used	15 [9-21]	20 [13-27]	17 [12-22]	8 [3-14]	17 [11-22]	15 [9-21]	17 [7-27]	40 [25-55]	18 [8-27]	7 [4-10]	8 [4-11]	7 [4-9]
Attendant used disposable gloves	3 [1-6]	2 [1-3]	3 [1-5]	3 [1-5]	8 [4-12]	7 [3-11]						
Plastic sheet used	6 [3-8]	9 [5-13]	7 [4-11]	**25 [20-30]**	**45 [40-50]**	**41 [36-46]**						
**Proportion of intrapartum and postnatal cord care in non-SBA home deliveries with 95%confidence intervals (%)**												
Thread/clamp used during delivery	98 [96-100]	99 [98-99]	98 [98-99]	94 [89-99]	97 [96-99]	97 [95-99]	79 [61-98]		79 [61-98]	99 [99-99]	98 [98-99]	99 [99-99]
Cord tied with boiled thread	11 [0-24]	14 [9-18]	12 [7-17]	52 [40-64]	60 [56-64]	59 [54-63]						
Cord cut with new/sterile blade	78 [62-93]	87 [82-91]	81 [75-87]	98 [97-99]	99 [99-99]	99 [98-99]	45 [37-54]		47 [38-55]	22 [17-27]	22 [17-28]	22 [17-27]
Nothing or only antiseptic applied to cord stump	92 [86-99]	91 [88-93]	91 [90-93]	**67 [53-81]**	**52 [42-62]**	**55 [44-66]**	76 [66-87]		76 [66-87]	50 [44-57]	49 [41-56]	52 [45-60]

Proportion of deliveries with protective antepartum and intrapartum practices for mothers who delivered at home without skilled birth attendance, separated by whether there was a traditional birth attendant (TBA) present. Values in **bold** font show practices where there was at least a 10 percentage point difference between TBA and non-TBA deliveries (whether positive or negative). The data for intrapartum TBA deliveries in Makwanpur was too sparse to calculate cluster-adjusted results.

**Table 7:** Proportion of deliveries with protective postnatal practices for mothers who delivered at home with or without a traditional birth attendant

**Care practice**	**Ekjut (Eastern India)**			**PCP (Bangladesh)**			**Makwanpur (Nepal)**			**Dhanusha (Nepal)**		
	**41%of home non-SBA deliveries were by TBA**			**83%of home non-SBA deliveries were by TBA**			**3%of home non-SBA deliveries were by TBA**			**23%of home non-SBA deliveries were by TBA**		
**Proportion of postnatal newborn care in non-SBA home deliveries with 95%confidence intervals (%)**												
	**No TBA**	**TBA**	**All non-SBA home**	**No TBA**	**TBA**	**All non-SBA home**	**No TBA**	**TBA**	**All non-SBA home**	**No TBA**	**TBA**	**All non-SBA home**
Clean cloth used for wrapping	77 [66-78]	82 [73-91]	79 [69-90]	97 [91-100]	99 [96-99]	98 [95-100]						
Immediate wiping (within 10 minutes)	22 [10-14]	23 [12-34]	23 [11-35]	**18 [9-26]**	**29 [16-51]**	**27 [15-39]**						
Skin-to-skin contact between mother and baby within 30 minutes	12 [5-8]	15 [6-24]	13 [5-21]	26 [8-44]	33 [15]	32 [20-44]						
Thermal care (wrapping or skin-to-skin contact within 10 minutes)	8 [1-4]	8 [2-13]	8 [2-15]	17 [7-27]	25 [8-33]	23 [7-39]	28 [17-40]	33 [12-54]	29 [17-40]	**66 [59-72]**	**56 [50-62]**	**65 [58-71]**
Delayed bathing (baby not bathed for at least 6 hours)	54 [47-58]	53 [46-60]	53 [46-61]	**61 [55-67]**	**75 [69-97]**	**72 [67-77]**	**12 [7-18]**	**31 [19-43]**	**13 [8-18]**	30 [23-37]	28 [22-35]	30 [23-37]
Colostrum not discarded							65 [56-75]	66 [46-86]	66 [56-75]	59 [53-66]	56 [48-63]	59 [52-66]
No pre-lacteal feed (breast milk first food)	72 [62]	68 [59-78]	71 [62-80]	74 [68-80]	83 [79-84]	81 [77-85]	**89 [84-94]**	**76 [56-97]**	**89 [84-94]**	43 [38-48]	39 [33-45]	42 [37-47]
Immediate breastfeeding (within 1 hour)	21 [10-14]	26 [14-39]	22 [11-34]	59 [49-70]	64 [34]	63 [53-74]	48 [36-61]	58 [36-80]	49 [36-61]	8 [6-11]	8 [6-11]	8 [6-10]
Only breast milk in first 24 hours	76 [65]	71 [64-78]	73 [67-80]	81 [73-90]	81 [77-80]	81 [76-86]						

Proportion of deliveries with protective postnatal care practices for mothers who delivered at home without skilled birth attendance, separated by whether there was a traditional birth attendant (TBA) present. Values in **bold** font show practices where there was at least a 10 percentage point difference between TBA and non-TBA deliveries (whether positive or negative).

In Eastern India, there was little difference in the coverage of ENC practices except for handwashing where TBA-attended deliveries had over twice the rate of handwashing than non-TBA births, although the rate of handwashing was still low (36%) (Table 6). TBA attendance was much rarer in both Nepali areas, but was associated with higher rates of delayed bathing, handwashing and CDK use, but lower rates of no pre-lacteal feeding in Makwanpur (Nepal). In Dhanusha (Nepal), TBA attendance did not appear to affect the coverage of ENC practices except that thermal care was somewhat lower for TBA attended deliveries (Table 7). TBA attendance seemed to have largest impact in Bangladesh, where rates of handwashing, plastic sheet use, immediate wiping and delayed bathing were all higher for TBA attended deliveries. However, TBA attended deliveries did report lower rates of applying something other than antiseptic to the cord stump. Considering the impact of TBA attendance as a whole, in all areas apart from Dhanusha (Nepal), there were much higher rates of handwashing among TBA-attended deliveries than for non-TBA attended ones. Reported attitudes to ENC among traditional birth attendants in rural southern Nepal [41] are consistent with observations in Mawkanpur (Nepal), but coverage is generally lower in TBA births in Dhanusha (Nepal).

Coverage of ENC practices in Bangladesh was generally higher for TBA deliveries than non-TBA deliveries but the coverage of ENC practices among home births with neither SBA nor TBA was typically still higher in Bangladesh than for other areas for: handwashing, plastic sheet use, cord tied with boiled thread (Table 6), skin-to-skin contact, delayed bathing and immediate breastfeeding (Table 7).

### Comparison of care practices between study areas

Overall, Bangladesh had the highest coverage of care practices across all delivery types, except for use of clean delivery kit and nothing or antiseptic applied to the cord.

### Hygiene

The coverage of hygienic practice varied widely between sites and delivery types (Figures 2 and 3, Table 4). Use of gloves, a plastic sheet and boiled thread to tie the cord was far from universal in Eastern India, while use of a clean delivery kit was not common in any study area. Cutting the cord with a sterile blade had good coverage everywhere except Dhanusha (Nepal) and home non-SBA births in Makwanpur (Nepal), but we note that in Nepal women were not asked if the blade was new; only if it had been boiled.

### Breastfeeding practices

Breastfeeding practices (Figure 4, Table 5) showed little variation across different delivery types compared to the other care practices. Eastern India, Bangladesh and Makwanpur (Nepal) had high coverage of no pre-lacteal feed and Bangladesh and Makwanpur (Nepal) also had moderate levels of immediate breastfeeding. Immediate breastfeeding was low in both Eastern India and Dhanusha (Nepal) and pre-lacteal feeds were common for non-institutional deliveries in Dhanusha (Nepal). Giving the newborn only breast milk in the first 24 hours was a common practice (70%-80%) in both Eastern India and Bangladesh, regardless of delivery type.

### Thermal care

**Figure 5:**
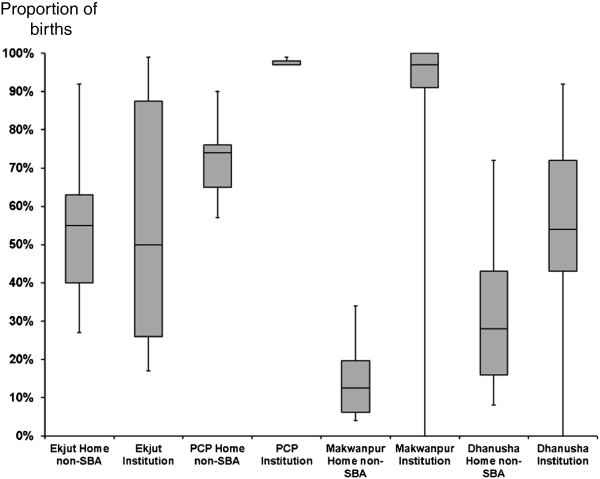
**Box plots of proportions of births within each control cluster for delayed bathing following home non-SBA and institutional deliveries across all study areas.** The bars show minimum and maximum ranges for the cluster proportions.

WHO recommends several practices related to thermal care [3] and the coverage of thermal care was generally the most variable between study areas and delivery types (Figure 4, Table 5). We analysed data on skin-to-skin contact, immediate wiping, wrapping and delayed bathing, all of which are known to protect newborn infants from hypothermia in the immediate postnatal period [42]. While delayed bathing was common (>80%) among home SBA and institutional deliveries in Makwanpur (Nepal), only 13% of infants born at home with no SBA were bathed after 6 hours. Bangladesh had the highest rates of delayed bathing across all delivery types, but both Bangladesh and Eastern India had low rates of skin-to-skin contact and immediate wiping. Dhanusha (Nepal) had much higher coverage of immediate wrapping (>60% for all delivery types) compared to other areas but lower levels of delayed bathing. Particularly for Eastern India and Dhanusha (Nepal), there is large variation between clusters in the coverage of delayed bathing (for all delivery types) compared to barely any variation in the Bangladesh clusters (Figure 5), suggesting that delayed bathing is a recognised, standard part of newborn care in Bangladeshi institutions.

## Discussion

We have described the coverage and wide variation of protective newborn care practices in large rural populations of South Asia. The provision of essential newborn care is far from universal even in institutional deliveries. We believe that there is considerable value in collating headline coverage figures based on thousands of births for essential newborn care in different settings. Detailed investigation of trends in care practices over time are beyond the scope of this paper but will be explored in further work.

Our results do have limitations: the studies cover different time periods, the definitions of skilled birth attendants vary slightly between studies, and the trials in Nepal captured slightly different (and less) information than those in Eastern India and Bangladesh.

An important limitation is that we cannot know how accurately a woman’s recollections of the birth reflect what happened, particularly when asking about the actions of an attendant such as hand washing or cord care. However, the recall period for such practices (six weeks) is shorter than in other surveys such as the DHS. There are also uncertainties in the timing of postnatal events such as wrapping or wiping and differences in how these questions were asked in the different studies. A recent study by Moran *et al*. [43] featuring over 160 birth narratives from Bangladesh and Malawi found that mothers were able to recall the sequence of events during childbirth and immediate newborn care practices regardless of delivery location or the timing of the survey relative to the birth, but that remembering the exact timing of event (e.g. wrapping within 30 minutes versus an hour) was more difficult. This suggests that answers to our questions about whether specific practices were followed or not were probably more accurate than answers about the timing of these practices. There may also be systematic differences between studies in how women answered the questions, either due to cultural differences or subtle differences in questionnaire design. We also do not have information on which births correspond to deliveries to the same mother, which could affect the observed proportions slightly.

Estimates are likely to be less reliable for home SBA and institutional deliveries, although for different reasons. Within each cluster there were relatively few home SBA births, leading to much variation between clusters, which is likely to be an artefact of these small numbers. While institutional deliveries were much less common than home non-SBA births, we nonetheless had several thousand institutional births for each study area. However, there was a much higher rate of missing data for these births, particularly for cord care practices. For instance, missing data for whether the cord was cut with a new or sterile blade ranged from 39% in Eastern India to 77% in Dhanusha (Nepal). Levels of missing data were also very variable between studies: for instance, data were missing for 77% of Bangladesh data on whether boiled thread was used to tie the cord compared to 32% for Eastern India. Both Eastern India and Makwanpur (Nepal) had low levels of missing data for hand washing in institutional deliveries but 58% of this data was missing for Bangladesh and 83% for Dhanusha (Nepal). In the case of Dhanusha the missing data is due to a skip sequence in the questionnaire, which meant that for most institutional deliveries these questions were not asked, particularly after 2009 when the questionnaire changed slightly. Nonetheless, we have at least 1285 records for care practices in institutional deliveries across all practices (up to almost 7000 records for breastfeeding practices). Thus we have included results from Dhanusha (Nepal) institutional deliveries, despite the high levels of missing data. In the other studies, it seems plausible that women giving birth might simply not know as much about what their attendant did as they would giving birth at home, but it also possible that these questions were not considered as relevant to institutional deliveries.

There will be inevitable differences in what an institutional and SBA delivery actually constituted between study areas. While the definitions are broadly the same, the training of the clinical staff and the equipment available is likely to differ by context.

Finally, many of the estimates have large confidence intervals due to a combination of genuine large variability in coverage and the effect of using cluster-level analysis techniques. These estimates should be considered as a guide to where certain practices have higher or lower coverage rather than as precise measurements.

Overall, coverage of protective care practices was highest for institutional deliveries, although coverage was considerably lower than 100% for all but a few practices. Potential messages for institutions across all study areas might be to improve immediate thermal care for the newborn and to encourage immediate and exclusive breastfeeding. The scarcity of skin-to-skin contact in institutional deliveries is consistent with a study of medical personnel in India [24]. A pilot study of the WHO Safe Childbirth Checklist program in Karnataka, India [17] found much lower baseline rates of sterile cord cutting and appropriate hand hygiene than in our study, but this study was carried out in a different state to ours, practices were recorded by independent observers and did not rely on mothers’ recollection. Thus our estimates of ENC coverage in institutions may be overly optimistic. Further work to understand why some of these practices are relatively uncommon in institutions is critical given recent increases in institutional deliveries in South Asia.

Home non-SBA deliveries show much variation both between study areas and care practices, but there is potential for large improvement in coverage of hand washing (Eastern India, Makwanpur (Nepal)) and simple thermal care (all study areas). Recent Demographic and Health Surveys for Nepal and Bangladesh show that there is still much room for improving the coverage of essential newborn care practices [21,22]. Promoting these relatively simple practices further could have great impact, given that most South Asian women in rural areas still give birth at home without an attendant [44] and this is likely to remain the case for the near future [19]. The variation between clusters within study areas suggests that health messages have been received differently in different communities. Low levels of immediate breastfeeding in Dhanusha (Nepal) are likely a result of cultural tradition to delay a baby’s first feed. Here, and in Eastern India, newborn infants are traditionally provided with a pre-lacteal feed of sugar water, honey or animal milk [45-47], while colostrum is discarded. Breastfeeding by the mother is frequently delayed until the third day of life.

Bangladesh shows much less variation between clusters for almost all practices and also has the highest coverage for many practices for all delivery types. Women giving birth at home without an SBA in Bangladesh are much more likely to have a traditional birth attendant present than in the other study areas, and this may in part explain the higher observed coverage of ENC practices in Bangladesh. However, the coverage of ENC in home non-SBA births remained relatively higher than the other study areas for Bangladeshi women giving birth without a TBA so this is unlikely to be the only explanation. Understanding how the higher coverage in Bangladesh has been achieved might provide valuable information for other South Asian contexts. In part this may reflect greater awareness in communities about preventive and primary care because of greater coverage of NGO programmes such as those run by the Bangladesh Rural Advancement Committee (BRAC), and also because women’s education levels were higher in the Bangladesh study areas than in the other areas. Certainly, the successes of BRAC and other community initiatives in Bangladesh have received a great deal of attention recently, including a recent Lancet series [48-52].

Other studies of essential newborn care practices in rural South Asia are broadly consistent with our findings, although most do not present coverage separately by delivery type. Darmstadt *et al*. reported lower levels of appropriate thermal care and hygienic cord care practices in the control arm of a community trial in rural Bangladesh [28], although their indicators of thermal care are not directly comparable to ours. The rates of reported immediate breastfeeding are consistent with our findings but a much higher proportion of deliveries reported a pre-lacteal feed [28]. Using 2007 DHS data, Shahjahan *et al.*[25] reported similar rates of breastfeeding within 1 hour and sterile cord care to our study in Bangladesh, but much lower rates of delayed bathing past 6 hours (50% vs 72-98%). Rahman *et al*. also examined newborn care practices using the 2007 Bangladesh DHS but only in non-institutional births in adolescents [53]. They reported very similar rates of cord and thermal care to the home non-SBA births in our Bangladeshi study but lower rates of immediate breastfeeding and no pre-lacteal feed. Syed et al. reported on newborn care among 3325 mothers from surveys taken between 2002 and 2004 in rural Bangladesh [26]. Results were not separated by type of delivery and showed generally lower levels of thermal care and early breastfeeding than in our study, but we note that our results are from a later time period.

Dhakal *et al.* reported on 150 births among women in rural Nepal [29], but these women were on average better educated than those in the studies considered here (literacy rate of 52% compared to 22-42% in our study areas). There was a higher rate of institutional delivery and higher rates of using a sterile blade to cut the cord among home births, although rates of CDK use at home were similar to our Nepalese study areas. Karas *et al*. reported on data from over 20,000 mothers in rural southern Nepal, in Sarlahi District, from a trial that ran between 2002-2006 [27]. Although observed ENC coverage was not reported separately for different delivery types, 90% of births took place outside of an institution and had no skilled birth attendance, thus reported rates can be seen as proxies for home non-SBA deliveries. The mothers had a similar literacy rate (26%) to those in our Nepali studies. Karas *et al*. [27] reported low rates of immediate wiping and wrapping, similar to our observations in Makwanpur (Nepal), but much lower than in Dhanusha (Nepal). The rate of delayed bathing was similar but cord cutting with a new blade and appropriate cord care were much more common than in either of our Nepali areas.

In a comprehensive but smaller scale study of newborn care practices in Haryana (India), separated by whether there was skilled attendance at birth, Upadhyay *et al*. also highlighted significant gaps in thermal care coverage and immediate breastfeeding for non-SBA births [18]. They also found that while coverage of ENC practices was higher for SBA deliveries, these were still generally far from universal, particularly for skin-to-skin contact (18%), immediate wiping (70%) and immediate breastfeeding (65%). However, reported coverage in SBA deliveries was generally higher than in our Eastern India study. We note that mothers included in the Upadhyay *et al.* study were on average better educated (76% literacy vs 28% in our study) and geographically distant from our study area (more than 1000 km). Nimbalkar *et al.* reported recently on newborn care practices among 150 families in rural Gujurat, India [30]. Only 6% of mothers had a home delivery and only 17% of mothers had no years of education (compared to 69% in our Indian study area). Across all deliveries, rates of handwashing, breastfeeding practices and thermal care were all much higher than in our Eastern India study, the latter two even when considering only institutional deliveries in our study. However reported use of kangaroo care (skin-to-skin contact) was still very low (18%).

Our data on a wide range of ENC practices, based on thousands of births across four different areas, highlights the continuing need to improve coverage of essential newborn care in rural South Asia, particularly among the poorest communities.

Culture shapes newborn care practices in the community, as well as thresholds for care seeking [54,55]. Intervention studies suggest that low-cost newborn care practices broadly supported by existing cultural norms can be further strengthened through community-based interventions. For example, studies from rural India have shown that families can underestimate newborns’ vulnerability to hypothermia in settings where many are born too small or too soon [42,56]. Similarly, in the plains of Nepal (Dhanusha) there is a traditional cultural belief that colostrum is harmful to the newborn and that no milk is available for the baby until the third day of life [57-59]. Also in Nepal, the mother and baby are considered ritually s‘polluted’ immediately after delivery and this belief may influence willingness to delay bathing of the baby [60]. In these contexts, community-based interventions in which lay facilitators, community health workers, mothers, and women’s groups discuss ways to keep newborns warm and more consistently has led to improvements in practices [36,61]. The intervention arms of the trials from which our data came from, where participatory action cycles were implemented with women’s groups, reported modest improvements in several such essential newborn care practices [61]. Other studies testing home visits by community health workers also reported increases in newborn care practices [13,56,62-66]. A combination of both of these strategies could be key to increasing the coverage of essential newborn care practices for women giving birth at home in South Asia.

The rising number of institutional deliveries throughout South Asia means that mothers and newborns are also increasingly exposed to the cultural and financial forces shaping care in local public and private health facilities. In rural India, studies have noted that the pressure of conducting many deliveries in short-staffed facilities can lead to an over-reliance on uterotonics, caesarean sections, and little time devoted to postnatal counselling [67]. Improving essential newborn care in such contexts is likely to require a strong focus on quality of care. Such initiatives might include implementation of the WHO Safe Childbirth Checklist Program [17], further support to WHO and UNICEF’s ‘Baby Friendly Hospital initiative’ [68], perinatal audits, empowering patient or community groups to demand better quality care, financial incentives for providers, training, and organizational management interventions [69]. While there are many studies documenting the implementation of these quality improvement strategies, a recent literature review found few good quality evaluations of their impact on maternal and newborn health outcomes in low and middle-income settings, making this is an important area of further research [70].

## Conclusions

There is a need to promote better newborn care after both institutional and home deliveries in rural South Asia. Good thermal care and infant feeding practices are a priority for institutions. For home deliveries, perinatal hygiene and handwashing, use of clean delivery kits and simple thermal care remain a problem. Many lives could be saved by mobilising families to improve these practices.

## Competing interests

The authors declare that they have no competing interests.

## Authors’ contributions

CP, SC and AP had the original idea for the study. CP and SC designed the data analysis in discussion with AP, MH, SR, AS, NS and CS. MH, SR, AS, NS and CS cleaned the data and performed the primary data analysis. CP performed the secondary data analysis for traditional birth attendants and prepared the tables and figures. NN, PT, KA, DM and AC are responsible for the trials. CP wrote the first draft of the paper and all authors read and commented on the draft. All authors read and approved the final manuscript.

## Pre-publication history

The pre-publication history for this paper can be accessed here:

http://www.biomedcentral.com/1471-2393/14/99/prepub

## Supplementary Material

Additional file 1 Relevant questions used for birth practice variables. Click here for file

Additional file 2 Definitions used for each delivery type in each study area. Click here for file
