# Supplementary material for: Is essential newborn care provided by institutions and after home births? Analysis of prospective data from community trials in rural South Asia
Source: BMC Pregnancy Childbirth. 2014 Mar 7;14:99. doi: 10.1186/1471-2393-14-99 (PMC4016384; doi:10.1186/1471-2393-14-99)
Supplement: Additional file 1 — Relevant questions used for birth practice variables. [file 1471-2393-14-99-S1.doc]

**Additional file 1**. Relevant questions used for birth practice variables. For multiple choice answers, the answers that would give a “Yes” to the birth practice are highlighted.

Pre-delivery hygienic birth practices, intrapartum and postnatal cord care practices.

| **Birth Practice** | **WHO definition of practice** (1) | **Study question** | | | |
| --- | --- | --- | --- | --- | --- |
|  |  | **PCP (Bangladesh)** | **Ekjut (eastern India)** | **Dhanusha (Nepal)** | **Makwanpur (Nepal)** |
| Attendant washed hands | Wash hands with soap and water before and after caring for a woman and newborn | Did the person wash his / her hands with soap before assisting you? | Did the person wash his / her hands with soap before assisting you? | Did the assistant wash their hands? | Did the person who helped, have washed his/her hands ? |
| Clean Delivery Kit used | (for home non-SBA births) Give mothers a disposable delivery kit and explain how to use it. | Was a safe delivery kit used during the delivery? | Was a safe delivery kit used during the delivery? | Did you use a safe delivery kit ? | Did you use this ? (on showing a clean delivery kit) |
| Attendant used gloves | Wear sterile or disinfected gloves when performing vaginal exam, delivery, cord cutting, repair of episiotomy, blood drawing | Were the following items used during delivery? **Disposable gloves**; thread/clamp; plastic sheet | Were the following items used during delivery? **Disposable gloves**; thread/clamp; plastic sheet | Not asked | Not asked |
| Plastic sheet used | Clean plastic sheet to place under mother | Were the following items used during delivery? Disposable gloves; thread/clamp; **plastic sheet** | Were the following items used during delivery? Disposable gloves; thread/clamp; **plastic sheet** | Not asked | Not asked |
| Thread / clamp used | Clamp and cut the cord | Were the following items used during delivery? Disposable gloves; **thread/clamp**; plastic sheet | Were the following items used during delivery? Disposable gloves; **thread/clamp**; plastic sheet | Did you tie the cord? | Did you tie cord ? |
| Nothing or only antiseptic applied to cord | Put nothing on the stump. *NOTE given a recent positive trial (2) using chlorhexidine we have included antiseptic in our definition. | What was put on the cord after it was cut? **Nothing**; Oil; **Antiseptic**; Vermillon; Mud; Talcum powder; turmeric; cloth; animal dung; ash/soot; other | What was put on the cord after it was cut? **Nothing**; Oil; **Antiseptic**; Vermillon; Mud; Talcum powder; turmeric; cloth; animal dung; ash/soot; other | What did you apply after cutting the cord? Oil; Turmeric;**medicine/Dettol** ; powder; soil/clay; ash; **nothing**; don’t know; other | What was put on the cord stump after it was cut? Oil; Turmeric; Unwashed cloth; washed cloth; **medicine/Dettol** ; powder; mud; **nothing**; don’t know; other |
| Cord cut with new/sterile blade | Make sure that instruments which penetrate  the skin (such as needles) are adequately  sterilized, or that single-use instruments are  disposed of after one use.  Thoroughly clean or disinfect any equipment  which comes into contact with intact skin | Was the instrument bought specifically for cutting the cord and unused? **Yes**; No; don’t know  --------  **OR**  Was the instrument boiled prior to cutting the cord? **Yes**; No; don’t know | What was the cord cut with after delivery? **New blade**; old blade; knife; scissor; other; don’t know  **And**  Was the instrument bought specifically for cutting the cord and unused? **Yes**; No; don’t know  --------  **OR**  Was the instrument boiled prior to cutting the cord? **Yes**; No; don’t know | What did you use to cut the cord? **a boiled blade?** Unboiled blade? Knife/scissors? Sickle/trowel? Bamboo? Don’t Know. Other. | Was the cord cut with: **a boiled blade?** Unboiled blade? Knife/scissors? Sickle/woodknife? Bamboo? Don’t Know. Other. |
| Cord tied with boiled thread | Cord ties (sterile) | Was the string used to tie the cord boiled prior to using? | Was the string used to tie the cord boiled prior to using? | Not asked | Not asked |

Postnatal newborn care practices

| **Birth Practice** | **WHO definition of practice** | **Study question** | | | |
| --- | --- | --- | --- | --- | --- |
|  |  | **PCP (Bangladesh)** | **Ekjut (eastern India)** | **Dhanusha (Nepal)** | **Makwanpur (Nepal)** |
| Kept Colostrum | Give your baby the first milk (colostrum). | Not asked | Not asked | Did you discard colostrum before feeding the baby first? | Did you throw first milk before you feed the baby first ? |
| Immediate breastfeeding (<1 hour) | Encourage initiation of breastfeeding within one hour of birth  And in first hour encourage the mother to initiate breastfeeding when baby shows signs of readiness. |  | When did you first put your baby to the breast? Never; **immediately**; **10-30 minutes**; **30 mins-1 hour**; 1-4 hours; >4 hours; don’t know | How long after the birth did you first feed you baby your milk? **Within 1 hour**; within 6 hours; 6-24 hours; within 2 days; within 3 days; within 4 days; after 4 days | How long after the birth did you first feed the baby? (in units of minutes, hours & days) **(<1 hour)** |
| Delayed bathing | DO NOT bathe the baby until at least 6 hours of age. | How long after birth was the baby bathed? **Baby not bathed**; immediately; within 6 hours; **7-24 hours**; **>24 hours**; Don’t know | How long after birth was the baby bathed? **Baby not bathed**; immediately; within 6 hours; **7-24 hours**; **>24 hours**; Don’t know | How long after the birth did you bathe the baby? <1 hour, 1-24 hours, **>24 hours** | How long after birth was the baby bathed? (in units of minutes, hours & days) **(>6 hours)** |
| No pre-lacteal feed | Do not give artificial teats or pre-lacteal feeds to baby. |  | What was the first food ever given to your baby? **Breast milk**; other | What did you feed the baby first of all? **Mother’s milk, Other mother’s milk**, Cow/buffalo milk, goat’s milk, formula milk/lactogen, tea or herbal water; Ghee; Oil; water; sugar / honey; honey; rich starch / litho/ rice porridge/ cerelec; fruit juice; rehydration fluids; medicine or vitamin syrup; other fluids; other; Don’t know | What was the first food given to the baby? **Mother’s milk, Other mother’s milk**, Cow/buffalo milk, Furmola/Lactozine, Milk food, Ghee/Sugar/Honey, Oil, Don’t know, Other |
| Thermal care^‡^ | Cover the baby and cover the head with a hat.  Leave baby on the mother’s chest in skin-to-skin contact  If the mother cannot keep the baby skin-to-skin because of complications, wrap the baby in a clean,  dry, warm cloth and place in a cot. Cover with a blanket. | How long after birth was the baby wrapped? Baby was not wrapped; **Immediately (<10 mins)**; 10-30 minutes; 30 mins-1 hour; 1-4 hours; >4 hours; don’t know  **OR**  How soon was the baby placed on the mother’s skin? Baby was not placed on skin; **Immediately (<10 mins)**; 10-30 minutes; 30 mins-1 hour; 1-4 hours; >4 hours; don’t know | How long after birth was the baby wrapped? Baby was not wrapped; **Immediately (<10 mins)**; 10-30 minutes; 30 mins-1 hour; 1-4 hours; >4 hours; don’t know  **OR**  How soon was the baby placed on the mother’s skin? Baby was not placed on skin; **Immediately (<10 mins)**; 10-30 minutes; 30 mins-1 hour; 1-4 hours; >4 hours; don’t know | How long after the birth did you wrap the baby in cloth? **Immediately**, within half an hour, after half an hour | How long after birth was the baby wrapped up? (in minutes and hours) **(<5 minutes)** |
| Clean cloth used for wrapping | Clean towels for drying and wrapping the baby | Was the baby wrapped with a washed or unwashed cloth? | Was the baby wrapped with a washed or unwashed cloth? | Not asked | Not asked |
| Skin-to-skin in 30 minutes | Leave baby on the mother’s chest in skin-to-skin contact.  And  Continue keeping the baby warm and in skin-to-skin contact with the mother. | How soon was the baby placed on the mother’s skin? Baby was not placed on skin; **Immediately (<10 mins)**; **10-30 minutes**; 30 mins-1 hour; 1-4 hours; >4 hours; don’t know | How soon was the baby placed on the mother’s skin? Baby was not placed on skin; **Immediately (<10 mins)**; **10-30 minutes**; 30 mins-1 hour; 1-4 hours; >4 hours; don’t know | Not asked | Not asked |
| Immediate wiping (<10 minutes) | Dry baby: immediately after birth, place the baby on the mother’s abdomen or on a warm, clean and  dry surface. Dry the whole body and hair thoroughly, with a dry cloth | How long after birth was the baby wiped? Baby not wiped; **Immediately (<10 mins);** 10-30 minutes; 30 mins-1 hour; 1-4 hours; >4 hours; don’t know | How long after birth was the baby wiped? Baby not wiped; **Immediately (<10 mins);** 10-30 minutes; 30 mins-1 hour; 1-4 hours; >4 hours; don’t know | Not asked | Not asked |
| Only breast milk in first 24 hours | Support exclusive breastfeeding.  DO NOT give any other feeds or water. | Have you given the baby anything other than breast milk since birth? Yes; **No**  **OR**  How old was the baby the first time you gave the baby something other than breast milk? 1 day; **2-7 days**; **1-3 weeks**; **4-6 weeks**; don’t know | Have you given the baby anything other than breast milk since birth? Yes; **No**  **OR**  How old was the baby the first time you gave the baby something other than breast milk? 1 day; **2-7 days**; **1-3 weeks**; **4-6 weeks**; don’t know | Not asked | Not asked |

**References**

1. WHO. Pregnancy, Childbirth, Postpartum and Newborn Care: a guide for essential practice [Internet]. 2006 [cited 2013 Jan 28]. Available from: http://www.who.int/reproductivehealth/publications/maternal_perinatal_health/924159084X/en/index.html

2. Arifeen SE, Mullany LC, Shah R, Mannan I, Rahman SM, Talukder MRR, et al. The effect of cord cleansing with chlorhexidine on neonatal mortality in rural Bangladesh: a community-based, cluster-randomised trial. Lancet. 2012 Mar 17;379(9820):1022–8.
