# Supplementary material for: Is essential newborn care provided by institutions and after home births? Analysis of prospective data from community trials in rural South Asia
Source: BMC Pregnancy Childbirth. 2014 Mar 7;14:99. doi: 10.1186/1471-2393-14-99 (PMC4016384; doi:10.1186/1471-2393-14-99)
Supplement: Additional file 2 — Definitions used for each delivery type in each study area. [file 1471-2393-14-99-S2.doc]

**Additional file 2**. Definitions used for each delivery type in each study area

| **Delivery Type** | **Definitions used in this study** | | | |
| --- | --- | --- | --- | --- |
|  | East India | Bangladesh | Nepal, Makwanpur | Nepal,  Dhanusha |
| Institution | Government hospital; charitable hospital; NGO facility; private facility. | Medical college hospital; district hospital; maternal and child welfare centre; upazilla health complex; union health and family welfare centre; NGO facility; Private facility; other hospital | Hospital; private hospital; primary health care; health post. | Janakpur or other hospital; NGO facility; Private facility; other hospital; Government health facility. |
| Home Skilled Birth Attendant (SBA) | Not in an institution and main attendant one of: Doctor or Nurse | Not in an institution and main attendant one of: Doctor; family welfare visitor; family welfare assistant; SBA; other outreach worker; midwife. | Not in an institution and main attendant one of: Doctor, Nurse, Auxiliary Nurse Midwife | Not in an institution and main attendant one of: Doctor, Nurse or Auxiliary Nurse Midwife |
| Home non-SBA | Not in an institution and not an SBA. Includes self, family, village doctor and traditional birth attendants. | Not in an institution and not an SBA. Includes self, family, village doctor and traditional birth attendants. | Not in an institution and not an SBA. Includes self, family and traditional birth attendants. | Not in an institution and not an SBA. Includes self, family, village doctor and traditional birth attendants. |
